# Oxytocin is implicated in social memory deficits induced by early sensory deprivation in mice

**DOI:** 10.1186/s13041-016-0278-3

**Published:** 2016-12-13

**Authors:** Jin-Bao Zhang, Ling Chen, Zhu-Man Lv, Xue-Yuan Niu, Can-Can Shao, Chan Zhang, Michal Pruski, Ying Huang, Cong-Cong Qi, Ning-Ning Song, Bing Lang, Yu-Qiang Ding

**Affiliations:** 1 Department of Histology and Embryology, Institute of Neuroscience, Wenzhou Medical University, Wenzhou, Zhejiang 325035 People’s Republic of China; 2 Key Laboratory of Arrhythmias, Ministry of Education of China, East Hospital, Shanghai, 200092 People’s Republic of China; 3 Department of Anatomy and Neurobiology, Collaborative Innovation Center for Brain Science, Tongji University School of Medicine, Shanghai, 200092 People’s Republic of China; 4 School of Medicine, Medical Sciences and Nutrition, Institute of Medical Sciences, University of Aberdeen, Foresterhill, Aberdeen, Scotland UK

**Keywords:** Sensory deprivation, Infraorbital nerve, Social memory, Oxytocin, Spatial memory, Autism

## Abstract

Early-life sensory input plays a crucial role in brain development. Although deprivation of orofacial sensory input at perinatal stages disrupts the establishment of the barrel cortex and relevant callosal connections, its long-term effect on adult behavior remains elusive. In this study, we investigated the behavioral phenotypes in adult mice with unilateral transection of the infraorbital nerve (ION) at postnatal day 3 (P3). Although ION-transected mice had normal locomotor activity, motor coordination, olfaction, anxiety-like behaviors, novel object memory, preference for social novelty and sociability, they presented deficits in social memory and spatial memory compared with control mice. In addition, the social memory deficit was associated with reduced oxytocin (OXT) levels in the hypothalamus and could be partially restored by intranasal administration of OXT. Thus, early sensory deprivation does result in behavioral alterations in mice, some of which may be associated with the disruption of oxytocin signaling.

**Electronic supplementary material:**

The online version of this article (doi:10.1186/s13041-016-0278-3) contains supplementary material, which is available to authorized users.

## Introduction

The rodent barrel cortex has proved to be a convenient model system for investigating neural plasticity in the cerebral cortex. The cytoarchitectonic units in layer IV, “barrels”, correspond one-to-one with the arrangement of whiskers on the contralateral snout in both morphology and function [1–4]. This unique topography largely benefits from developmental fine-tuning elicited by sensory inputs from the whiskers. For example, sensory deprivation before postnatal day 4 (P4), either by lesioning elements of the afferent pathway or by ablating, plucking, or trimming the whiskers, can disrupt the formation of the barrels [5–8] and alter the physiological properties of neurons in the barrel cortex [9, 10]. Moreover, somatosensory experience re-organizes the cortical plasticity both during perinatal development and adulthood [11–13]. In addition, plasticity of cortical neurons are also changed after sensory deprivation, for example layer IV neurons rapidly diminishes synaptic plasticity after sensory deprivation [14, 15]. Sensory experience also affects dendritic protrusions and local circuitry [16–20] suggesting possible implication in the process of learning and memory.

Our previous finding has shown that callosal axons of layer II/III pyramidal neurons in the somatosensory cortex cross the midline region and then find correct target areas in the opposite hemisphere to establish callosal connections during the early postnatal days, and this process depends on spontaneous neuronal activity [21]. In addition, callosal projections in the somatosensory cortex can be disrupted by unilateral transection of ION, and this disruption is permanent in adult brain, showing that early sensory input is also critical for the formation of inter-hemispheric connection in the cortex [22]. We thus hypothesized that the abnormal development of brain circuits caused by the early sensory deprivation may lead to alterations of adult behaviors. Unfortunately, although the behavioral deficits in the sensory deprivation model have been explored before, most of them are limited to whisker-related behaviors [23–25].

OXT is mainly synthesized in the hypothalamic paraventricular (PVN) and supraoptic nuclei. For neuroendocrine functions, OXT is transported via neurosecretory axons to the posterior hypothalamus, stored in the pituitary gland and released into the peripheral bloodstream [26]. In the central nervous system, OXT-positive neurons project widely and their receptors are distributed in many regions of the brain, including the olfactory bulb, lateral septum, hippocampus and amygdala [27]. The role of OXT has well been established in social, maternal and sexual behaviors, and dysfunction of OXT system has been considered to be a factor in the etiology of autism [28–32]. A recent study showed that the synthesis and secretion of OXT in the hypothalamus are reduced significantly after bilateral whisker deprivation from P0 to P14 [33]. However, whether this is the case for unilateral sensory deprivation remains unknown. And it is also unclear whether the reduction of OXT is a transient event occurring only in postnatal development or a permanent defect persisted in adulthood. In addition, so far there is no data available which connect adult social behaviors, OXT expression and early sensory deprivation.

Here, we investigated the behavioral manifestation of adult mice subjected to early sensory deprivation by unilateral transection of ION at P3. ION-transected mice showed reduced social memory and defective spatial memory. Interestingly, the ION mice presented decreased OXT levels in the hypothalamus at P14 and in adulthood, and OXT supplementation restored their social memory deficit. Our results indicate that early sensory deprivation indeed results in alterations of adult behaviors in mice, and reduced OXT may be implicated in social memory deficits.

## Methods

### Mice

C57BL/6J mice were used in this study. After weaning at postnatal day 20–25, all mice were group-housed by gender in standard plastic cages (4 or 5 per cage) with food and water available ad libitum. Mice were maintained in a temperature and humidity controlled room (22 ± 1 °C and 50–60% relative humidity), with a 12-h light/12-h dark cycle (lights on at 7:00 AM). All animal care was in accordance with the Tongji University Ethics Committee on Animal Studies and experimental protocols were reviewed and approved by the Animal Research Committee of Tongji University School of Medicine, Shanghai, China.

### Experimental design

Unilateral transection of ION was conducted at P3 as described previously [5, 34]. Under anesthesia on ice, unilateral ION on either side was exposed and about 3 mm ION was cut off. For every litter, pups were distributed equally to three groups (Normal: naive mice; Sham: sham-operated mice with operation but without ION transection; ION: mice with ION transection). Both male and female mice were used in all the tests (male:female≈1:1 in each group). Behavioral tests were commenced at P60 and finished within 45 days. Since the naive mice and the sham-operated mice showed no significant difference in all the behavioral tests, we just used the sham group as a control in following studies: immunostaining and enzyme-linked immunoassay of OXT and AVP, and rescue experiment using OXT in social memory test. The three chamber test was conducted at P65 to assess the effect of OXT on the social memory of ION mice.

### Behavioral tests

Behavioral tests were conducted in a sound proof room with a neutral environment, in the following order: open field test, novel object recognition test, two-trial direct interaction test, three chamber test, dark-light exploration test, elevated plus maze test, Rota-rod test, olfactory habituation/dis-habituation test, and Morris water maze test. After each test, mice were returned to their home cages and given 2–3 day of rest between the two tests. All tests were conducted during the light phase of the light/dark cycle. Mice were given a 30-min habituation period after transportation to the behavioral room before the start of each test. The experimenter was blind to the group identity of the tested mice. Most behavioral tests were videotaped with a video camera and the recorded video file was analyzed by a trained researcher.

#### Open field test

The open field test was performed to analyze the locomotor activity and anxiety-like behaviors of rodents in a novel environment [35]. The open field apparatus (Med Associates, Inc.) was a transparent plexiglass box (30 cm × 30 cm × 21 cm) placed in a soundproof box with controlled lighting (about 200 lx). Mice were placed along the edge of the arena and allowed to freely explore it for 30 min. Average velocity, total distance travelled, distance travelled in the central zone (10 cm × 10 cm square area) and time spent in the central zone were recorded by Activity Monitor software (Med Associates, Inc.).

#### Novel object recognition test

The novel object recognitiontest was conducted in a big soundproof box (60 cm × 60 cm × 100 cm), containing a square, high-walled arena (25 cm × 25 cm × 25 cm) at its bottom. The light intensity inside the arena was about 75 lx. The test was performed as described previously [36]. Briefly, it consisted of three phases: habituation sessions (day 1 and day 2, no object inside, 20 min/day), training session (day 3, two identical objects placed symmetrically, 10 min), and test session (1 h after training, one familiar object replaced by a novel one, 5 min). The two kind of objects used in the test have similar size and smell but differ in shape and texture.

The time spent exploring each object was scored when the subject was sniffing towards the object within 2 cm. Discrimination index was defined as [(time spent exploring the novel object – time spent exploring the familiar object)/time spent exploring the novel object and the familiar object] × 100%.

#### Two-trail direct interaction test

This social memory test was conducted as described previously [37, 38] with minor modifications. It was conducted in a non-transparent, white open field arena. One day before the test, all the test mice were habituated to the arena for 20 min. On the test day, after another 5 min of habituation, the test mouse was allowed to investigate a novel stimulus mouse (age- and gender-matched C57BL/6 J mice) for 5 min (trail 1). At the end of trial 1, we removed the stimulus animal and returned it to an individual holding cage. After an interval of 1 h, the test was run again with either the previously encountered mouse or another novel mouse (trail 2). The following behaviors were scored as social interaction: anogenital and nose-to-nose sniffing, following (within 2 cm) and allogrooming. Any aggressive encounters between animals lead to the terminating of the experiment and exclusion of the data from the analysis.

#### Three chamber test

The three chamber social apparatus was a (90 cm × 50 cm × 30 cm) plexiglass box, which was divided into three equal size compartments by two transparent partitions. At the floor level of each partition, there was a square opening (5 cm × 5 cm) located in the center, allowing access into each chamber. Two small, round wire cages were put in the diagonal corner of the apparatus for enclosing a stranger mouse or a similar-size ball and two weighted bottles were placed on the top of the cages to prevent the test mice from climbing over the wire cages.

The test was designed in accordance with previous studies [39, 40]. The day before the test, all the test mice were habituated to the apparatus for 20 min with the two empty cages inside and all the stranger mice were habituated inside the wire cages for 20 min at a separate time. On the test day, after a 10-min habituation period, a stranger mouse was placed into one of the two wire cages while an inanimate ball was placed into the other. Then, the subject mouse was placed in the center, and allowed to freely explore the chamber for 5 min. The subject had the choice between a stranger mouse and an inanimate ball in this phase (sociability phase). Immediately after the sociability phase, the inanimate ball was replaced by a second stranger mouse, and the subject was allowed to freely explore the chamber for another 5 min. Thus, the subject would now have the choice between the mouse that the subject had already encountered and a new stranger mouse (preference for social novelty phase). After an interval of 30 min, the second stranger mouse was replaced by a third stranger mouse, and the subject was allowed to freely explore the chamber for 5 min again. Now it had the choice between familiar stranger 1 that it had already encountered 30 min before and the unfamiliar stranger 3 (social memory phase). Interaction time was scored when the subject was sniffing towards the cages within 2 cm. The location of the stranger 1 was alternated between tests. All the stranger mice used in the test were age- and gender-matched C57BL/6 J mice which were never exposed to the subjects before.

#### Dark-light exploration test

The Dark-light exploration test assessed the anxiety-like behavior of rodents [36]. The apparatus was a (45 cm × 20 cm × 20 cm) plexiglass box divided into two parts: 1/3 was painted black, covered by a lid, and 2/3 was open. The two compartments were separated by a wall containing an opening (5 cm × 5 cm) at floor level. The light intensity was about 500 lx in the opened part. Mice were placed in the center of the dark compartment (facing away from the opening) and allowed to explore the apparatus for 5 min. The time spent in the light box and the numbers of entries into the light box were recorded.

#### Elevated plus maze

This test assessed the anxiety-like behavior of rodents [36]. The apparatus was a plus-shaped maze which was elevated 40 cm above the ground and consisted of two enclosed arms (30 cm × 5 cm, with 15 cm high nontransparent walls) and two open arms (30 cm × 5 cm). The test was conducted in a testing room with standard lighting (about 100 lx). Each mouse was placed in the center (5 cm × 5 cm) of the maze facing one of the enclosed arms and allowed to explore the apparatus for 5 min. Time spent in the open arms and the numbers of entries into the open arms were measured.

#### Rota-rod test

The Rota-rod test was used for the assessment of motor coordination and motor learning as described previously [36, 41]. The day before the test, all mice were habituated in the Rota-rod apparatus (Ugo Basile) for 5 min with a constant speed (4 rpm). From day 1 to day 5, the test was performed two times (5 min, 2 h interval) with 4–40 rpm accelerating speed. The latency to fall off the apparatus was measured and the average of the two trials was calculated.

#### Olfactory habituation/dis-habituation test

The olfactory habituation/dishabituation test was performed as described previously [39, 42]. All the test mice were singly housed in a clean home cage during the test, with a cotton swab positioned 9 cm above the bedding. After a 30 min habituation period (with a dry cotton swab presented), the test mice were allowed to investigate different odors: water, orange extract (1:100 dilution), and urine (collected from unfamiliar C57BL/6 J male mice, 1:100 dilution). The odors were sequentially presented in the following order: water 1, water 2, water 3, orange 1, orange 2, orange 3, urine 1, urine 2, and urine 3. Each odor was presented for 2 min, with a time interval of 1 min between each subsequent presentation. Time spent sniffing the odor was measured by manual observation with a stopwatch. Time was only scored when the test mouse was sniffing the swab within 2 cm.

#### Morris water maze test

This test was used to evaluate spatial learning and memory in rodents according to the method of Voorhees and Williams [43]. It was conducted in an open circular pool (1.2-m diameter) filled with opaque water of 22 ± 1 °C. The maze was divided into four hypothetical and equal quadrants and a hidden platform was positioned in the middle of the target quadrant 2 cm below the water surface. Movement of the mice was recorded by a camera and further analyzed by off-line video tracking software (EthoVision XT 8.0, Noldus Technology). During the acquisition phase, mice were trained to find the hidden platform. Four trials were conducted per day for 5 consecutive days. Mice were placed into a quadrant located either left, right, or opposite to the target quadrant from a semi-random set of start locations, with the restriction that one trial each day is started from each of the four positions. A mouse that failed to find the platform within 60 s was guided to it. All mice were left on the platform for 25 s in each trail. Latency to find the platform was measured automatically by the software. The probe phase was performed 24 h after the final training trial. The hidden platform was then removed from the pool, and each mouse was allowed to explore the maze for 60s. Average velocity and time spent in the target quadrant were measured automatically by the software.

### Immunohistochemical staining

Mice were deeply anesthetized with sodium pentobarbital (100 mg/kg bodyweight) and intracardial perfusion was performed with phosphate-buffered saline (PBS; pH7.4) followed by 4% paraformaldehyde (PFA) in PBS. The brains were removed immediately, post-fixed overnight in PFA, and cryoprotected in 30% sucrose in 0.1 M PBS for 36–48 h. Thirty μm-thick coronal brain sections were cut serially on a cryostat (Leica). The sections were immunostained for OXT or VP with the avidin-biotin complex methods (Vector Labs). After Antigen retrieval in sodium citrate solution at 95°C [44], sections were incubated with the primary antibody in 0.01 M PBS containing 0.3% Triton X-100 and 1% normal horse serum overnight at 4 °C. After washes, sections were incubated with a biotinylated goat anti-rabbit secondary antibody (1:500; Vector Laboratories) for 2 h at room temperature, followed by the application of avidin-biotin-peroxidase (1:200; Vectastain Elite ABC kit, Vector Laboratories) for 1 h at room temperature. Immunoreactivity was visualized with the diaminobenzidine Substrate Kit (Vector Laboratories). For primary antibodies, we used rabbit anti-OXT (1:10,000; Millipore) and rabbit anti-VP (1:10,000; Millipore). Images of every fourth coronal section throughout the entire PVN regions were collected using a 10× objective under a Nikon Eclipse 80i microscope according to the mouse brain in stereotaxic coordinates (45) and analyzed in a blinded fashion using ImageJ software. Numbers were multiplied by four to provide an estimate of the total number of positive cells.

### Enzyme-linked immunoassay analyses

Enzyme-linked immunoassay analyses were performed to quantify levels of OXT and VP in the brain. The hypothalamus or the whole brain was quickly removed and frozen in liquid nitrogen. The brain region of the hypothalamus was determined by the mouse brain in stereotaxic coordinates [45]. We cut brain into slices (2–3 mm anterior to the optic chiasm and at posterior border of mammillary body), and the brain slice between the two contains the whole rostrocaudal extent of PVN and supraoptic nucleus, the two major sites of OXT-containing neurons in the brain. All samples were stored at −80 °C until assayed. Neuropeptides were extracted as described previously [46]. Briefly, tissues were sonicated in ice-cold distilled water containing a protease inhibitor cocktail (Complete Mini, Roche), incubated at 95 °C for 10 min, extracted by addition of HCl and acetic acid to a final concentration of 0.02 M and 0.1 M respectively, and centrifuged for 20 min at 12,000 rpm. OXT and VP concentrations were measured using EIA kits (Pheonix Pharmaceutics, EK-051-01 for OXT and EK-065-07 for VP), according to the manufacturer’s instructions. The final result was corrected by the total protein concentration of the supernatant, which was measured using the BCA assay (Pierce).

### OXT administration

All the mice were well habituated to the environment and the social apparatus before the three chamber test was performed. Twenty min before the test began, mice were intranasally given a volume of approximately 5μl of isotonic saline or OXT dissolved in isotonic saline (200μg/kg; Pheonix Pharmaceutics) as described previously [47, 48].

### Statistical analyses

Statistical analyses were performed using IBM SPSS Statistics 19 software. Differences among multiple groups were compared using one-way ANOVA, with Fisher’s LSD post hoc analysis. For enzymes-linked immunoassay and immunostaining experiments, 2-tailed Student’s *t*-test was used. Data from the direct interaction test, three chamber test, Rota-rod test, olfactory habituation test, and the acquisition phase of the Morris water maze were analyzed by repeated measures ANOVA, followed by Fisher’s PLSD post hoc test to compare group means when a significant F-value was determined. All data were expressed as mean ± SEM. Results were considered significant when corrected *p*-value <0.05.

## Results

**Fig. 1:**
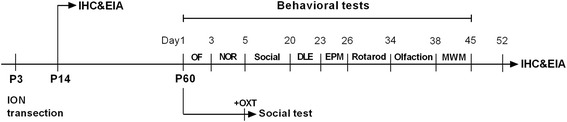
The schedule of the experimental design. DLE, dark-light exploration test; EIA, Enzyme-linked immunoassay analyses; EPM, elevated plus maze test; IHC, immunohistochemistry staining; NOR, novel object recognition test; Olfaction, olfactory habituation/dis-habituation test; OF, open field test; Social, two-trial direct interaction test and three chamber test; Rotarod, Rota-rod test; MWM, Morris water maze test

To demonstrate the behavioural manifestation of ION-transected mice, a spectrum of behavioural tests have been designed and performed as depicted in Fig. 1.

### Normal locomotor activity, motor coordination and anxiety-like behaviors in ION-transected mice

**Fig. 2:**
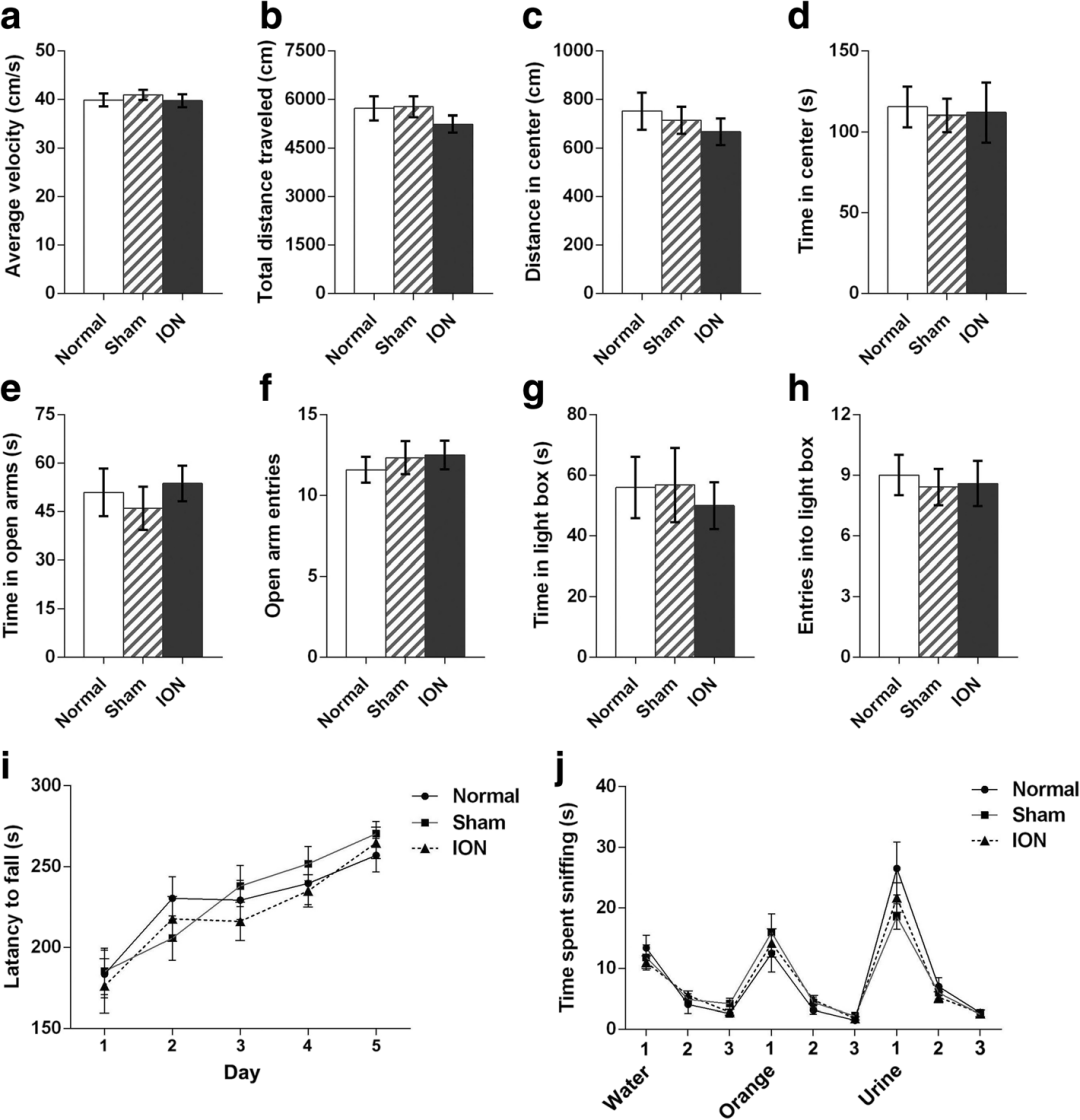
Behavioral data from open field test, elevated plus maze test, dark-light exploration test, rota-rod test and olfactory habituation/dis-habituation test. **a**–**d** Data of Open field test. Average velocity (**a**), total distance traveled (**b**), distance traveled in the central zone (**c**) and time spent in the central zone (**d**). **e**, **f** Data of elevated plus maze test. Time spent in the open arms (**e**) and number of entries into open arms (**f**). **g**, **h** Data of dark-light exploration test. Time spent in the light box (**g**) and number of entries into the light box (**h**). **i** Latency to fall off the apparatus in the accelerating Rota-rod test. Data were obtained from two trials per day during 5 consecutive days. All groups improved motor performance over days and no significant group effect was detected. **j** Time spent sniffing a sequential presentation of nonsocial odors (water, orange) and social odors (urine) in the olfactory habituation/dishabituation test. All groups showed significant habituation and dishabituation to non-social and social odors. Data from (**a**–**h**) were analyzed using one-way ANOVA and no significant differences were detected across groups. Data from (**i**, **j**) were analyzed using repeated-measures ANOVA. Results are expressed as mean ± SEM. 6 M (males) and 6 F (females) in Normal; 5 M and 8 F in Sham; 6 M and 7 F in ION group (**a**–**d**). 5 M and 7 F in Normal; 6 M and 6 F in Normal; 5 M and 7 F in ION group (**e**–**h**). 5 M and 7 F in Normal; 6 M and 6 F in Sham; 7 M and 5 F in ION group for (**i**). 5 M and 6 F in Normal; 6 M and 6 F in Sham; 5 M and 6 F in ION group for (**j**)

Consistent with previous reports [5–8], P3 unilateral transection of ION resulted in a loss of barrels in the contralateral cortex (Additional file 1: Figure S1). We first performed open field test to demonstrate the spontaneous locomotor activity in the ION mice. No significant differences were detected across the three groups in average velocity (F_2,36_ = 0.280, *p* = 0.758; Fig. 2a) and total travelled distance (F_2,36_ = 0.842, *p* = 0.439; Fig. 2b), indicating normal locomotor activity in ION mice. Moreover, there were no significant differences in distance travelled in the central zone (F_2,36_ = 0.451, *p* = 0.640; Fig. 2c) and time spent in the central zone (F_2,36_ = 0.036, *p* = 0.965; Fig. 2d), suggesting that the basal anxiety level did not differ across groups. To further confirm this, we conducted two other tests, the elevated plus maze test and the dark-light exploration test, which are widely used for assessing anxiety-like behaviors in rodents [35, 36]. No significant differences were observed in both of the two tests, as shown by a similar time spent in the open arms (F_2,33_ = 0.353, *p* = 0.706; Fig. 2e) and numbers of entries into the open arms (F_2,33_ = 0.287, *p* = 0.752; Fig. 2f) in the elevated plus maze test, and a similar time spent in the light box (F_2,33_ = 0.131, *p* = 0.878; Fig. 2g) and numbers of entries into the light box (F_2,33_ = 0.089, *p* = 0.915; Fig. 2h) in the dark-light exploration test.

In the Rota-rod test, the repeated-measures ANOVA (3 groups × 5 days, with repeated measures on days) revealed that there was no significant effect of group on the latency to fall off (group: F_2,33_ = 0.242, *p* = 0.786; group × trial: F_8,132_ = 0.804, *p* = 0.600; Fig. 2i), and all groups showed improved motor performance over days (F_4,132_ = 27.824, *p* < 0.001; Fig. 2i). The data sorted by sex are shown in Additional file 2: Table S1. The results demonstrate that the ION mice have normal motor coordination and motor learning.

### Normal olfaction in ION-transected mice

We then assessed the olfactory functions of ION-transected and control mice. As presented in Fig. 2j, all groups showed significant habituation to both nonsocial and social odors, and exhibited a decline in time spent sniffing the swabs with sets of identical odors (water: F_2,62_ = 50.82, *p* < 0.001; orange: F_2,62_ = 50.33, *p* < 0.001; urine: F_2,62_ = 102.06, *p* < 0.001; no group interactions). Meanwhile, all groups showed a significant dis-habituation between odors, as shown by an increase in time spent sniffing the first swab of a new odor compared with the last swab of the previous odor (water 3-orange 1: F_1,32_ = 53.09, *p* < 0.001; orange 3-urine 1: F_1,32_ = 132.50, *p* < 0.001; no group interactions). The data sorted by sex are shown in Additional file 2: Table S1. These results suggest that ION mice have normal olfaction.

### Social memory deficit in ION-transected mice

**Fig. 3:**
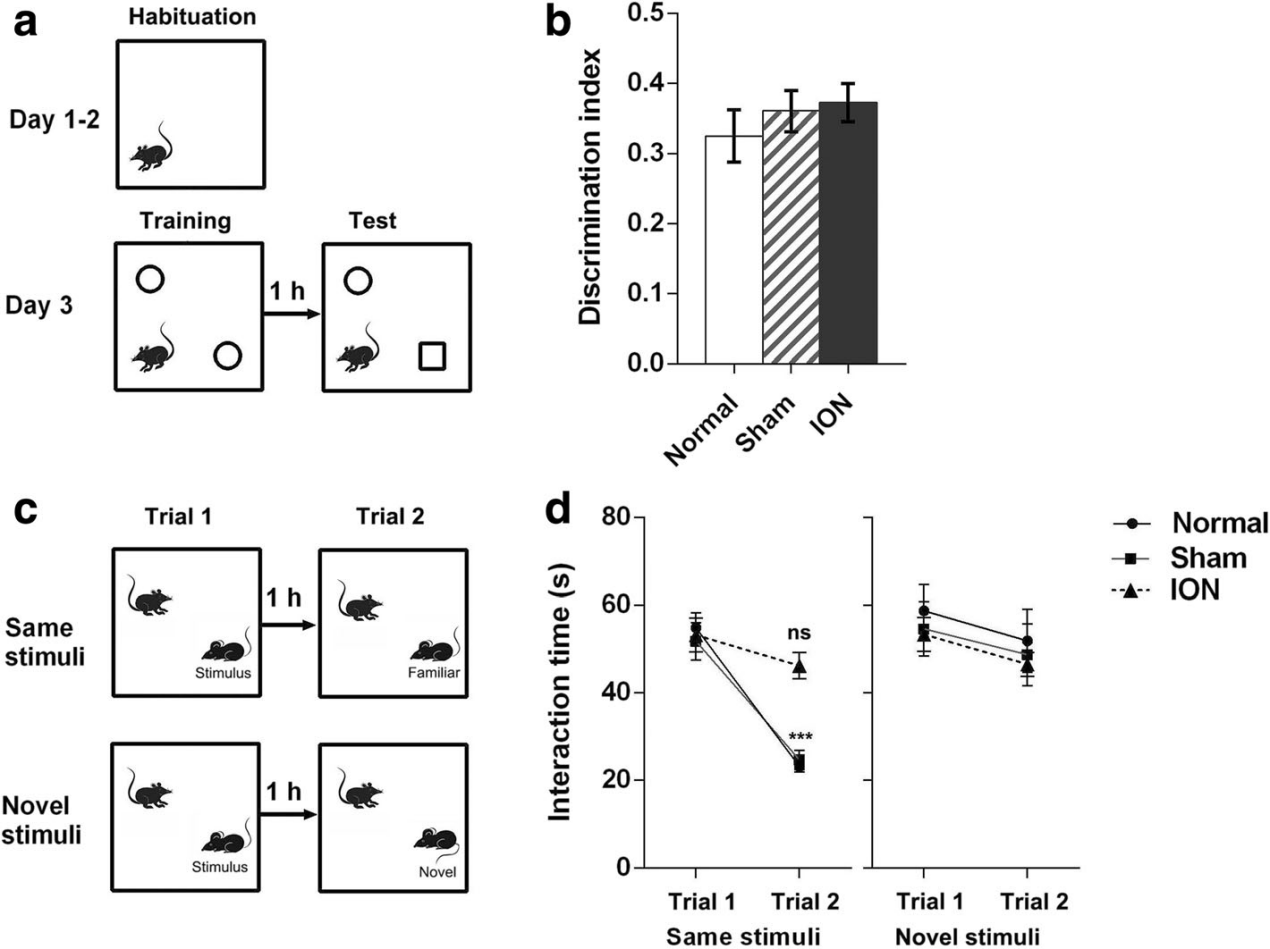
Behavioral data from novel object recognition test and two-trial social memory test. **a** Schematic diagram of the novel object recognition test. **b** Novel object discrimination index in the novel object recognition test. **c** Schematic diagram of the two-trial direct interaction test. **d** Left: two-trial direct interaction test using the same stimulus mouse in the two trails. Naive mice and sham-operated mice showed decreased interaction time in trial 2 compared with in trial 1, while ION mice showed a similar interaction time (*p* = 0.139). Right: two-trial direct interaction test using different stimulus mice in the two trails. All groups showed a similar interaction time in the two trails. Data from the NOR test were analyzed by one-way ANOVA and data from the two-trial direct interaction test were analyzed by repeated-measures ANOVA (***p < 0.001 via Fisher’s PLSD post hoc tests). 6 M (males) and 6 F (females) in Normal; 5 M and 6 F in Sham; 6 M and 6 F in ION group (**b**). Six M and 6 F in Normal; 5 M and 7 F in Sham; 6 M and 6 F in ION group for *left panel* (**d**); 4 M and 4 F per group for *right panel* (**d**)

We first examined object memory in the ION mice using the novel object recognition test (Fig. 3a) and no significant differences were detected across groups on discrimination index (F_2,32_ = 0.634, *p* = 0.537; Fig. 3b). Mice in all groups spent more time exploring the novel object compared with the familiar one (discrimination index > 0). Thus, ION-transected mice have normal memory of inanimate objects.

In contrast, the ION mice showed reduced memory to a familiar mouse in the two-trial direct interaction test. Repeated-measures ANOVA revealed a significant interaction between group and trial (F_2,33_ = 8.798, *p* < 0.001, Fig 3d, left) when encountering the same stimulus mouse in the two trials (upper panel in Fig. 3c). The naive mice and sham-operated mice had a decline in investigation time in the two trials (trial 1 to trial 2, Normal: F_1,11_ = 68.54, *p* < 0.001; Sham: F_1,11_ = 28.89, *p* < 0.001), but the ION mice showed similar investigation times (trial 1 to trial 2, ION: F_1,11_ = 291.07, *p* > 0.05). In addition, all groups showed similar investigation times during trial 1 and trial 2 when two different mice (lower panel in Fig. 3c) were encountered in the two trials (ANOVA, trial: F_1,21_ = 3.755, *p* = 0.066; group × trial: F_2,21_ = 0.009, *p* = 0.992; Fig. 3d, right), illustrating that the reduced memory to a familiar mouse is not caused by lowered sociability of ION mice.

**Fig. 4:**
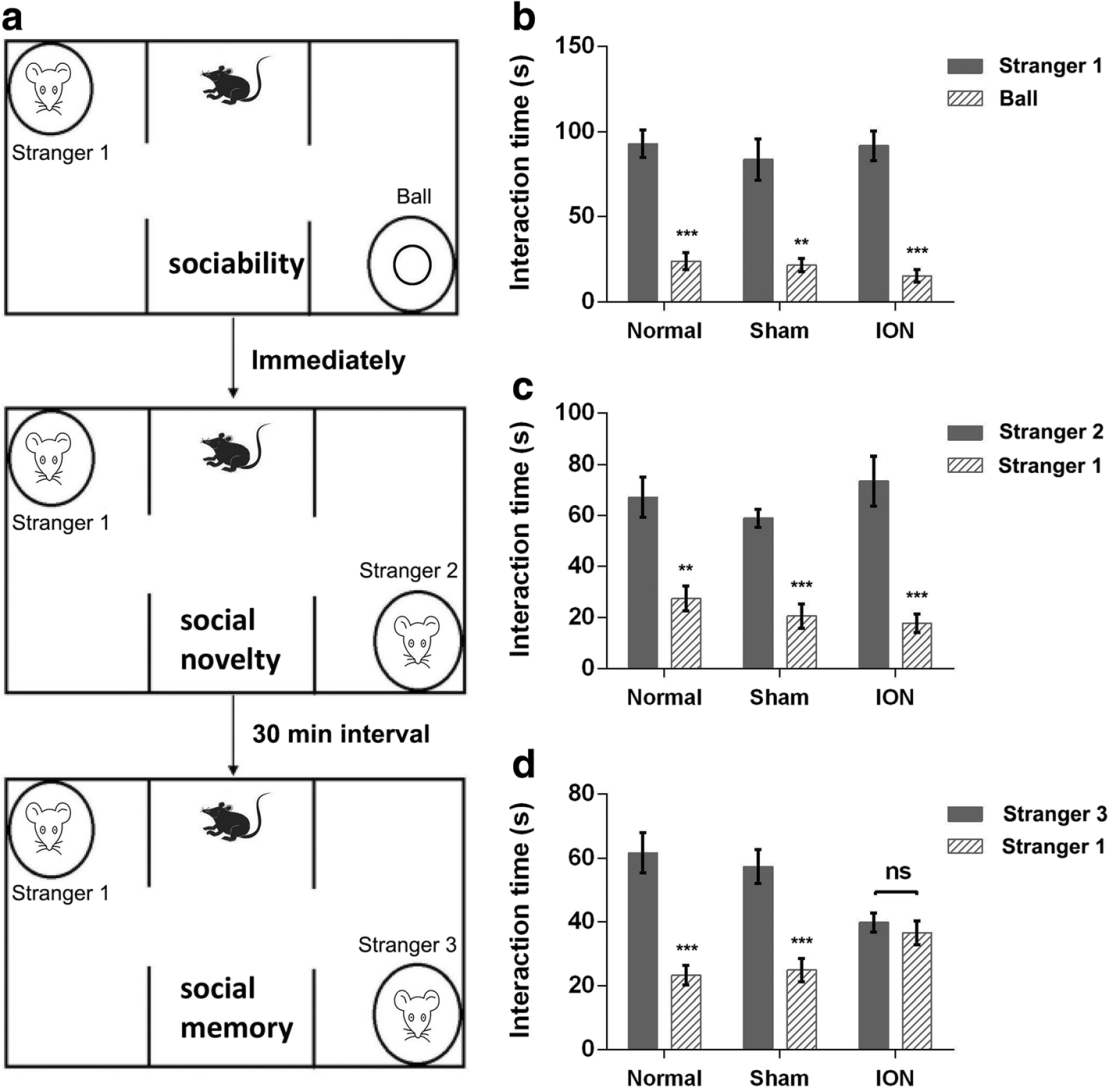
Behavioral data from three chamber test. **a** Schematic diagram of the three chamber test. **b** Sociability of mice in the three chamber test. All groups showed preference for a stranger mouse vs an inanimate ball. **c** Social novelty in the three chamber test. All groups showed preference for a novel stranger vs a familiar stranger. **d** Social memory in the three chamber test. Naive mice and sham-operated mice showed preference for a novel stranger vs a familiar stranger encountered 30 min ago, whereas ION mice showed similar investigation time (*p* = 0.421). Data were analyzed by repeated-measures ANOVA (**p < 0.01, ***p < 0.001 via Fisher’s PLSD post hoc tests). Results are expressed as mean ± SEM. 7 M (males) and 4 F (femals) in Normal; 5 M and 6 F in Sham; 6 M and 6 F in ION group

To further assess the social behaviors of ION-transected mice, we conducted the three chamber social approach test with the design shown in Fig. 4a. In the first phase, the test mice were allowed to freely explore an inanimate ball and an animate stranger 1. All groups demonstrated a significant preference for exploring stranger 1 and no significant group effect was observed (side: F_1,31_ = 142.34, *p* < 0.001; group: F_2,31_ = 0.359, *p* = 0.735; group × side: F_2,31_ = 0.512, *p* = 0.604; Fig. 4b). Immediately after the sociability test, the test mice were allowed to freely explore a novel stranger 2 and the now-familiar stranger 1. All groups showed similar preference for social novelty (side: F_1,31_ = 72.837, *p* < 0.001; group: F_2,31_ = 0.815, *p* = 0.452; group × side: F_2,31_ = 1.167, p = 0.325; Fig. 4c). After a 30 min interval, we performed the test again, with stranger 2 replaced by a novel stranger 3. A significant interaction was found between group and side in this phase (F_2,31_ = 12.246, *p* < 0.001; Fig. 4d). Fisher’s PLSD post hoc analysis revealed that the naive mice and the sham-operated mice spent more time investigating the novel stranger 3 than the familiar stranger 1 (Normal: F_1,10_ = 27.972, *p* < 0.001; Sham: F_1,10_ = 44.504, *p* < 0.001), whereas the ION mice showed similar investigation times (ION: F_1,11_ = 0.698, *p* > 0.05). The data sorted by sex are shown in Additional file 2: Table S1. These results illustrated that ION-transected mice have normal sociability and preference for social novelty, but deficit in the social memory.

### Defective spatial memory in ION-transected mice

**Fig. 5:**
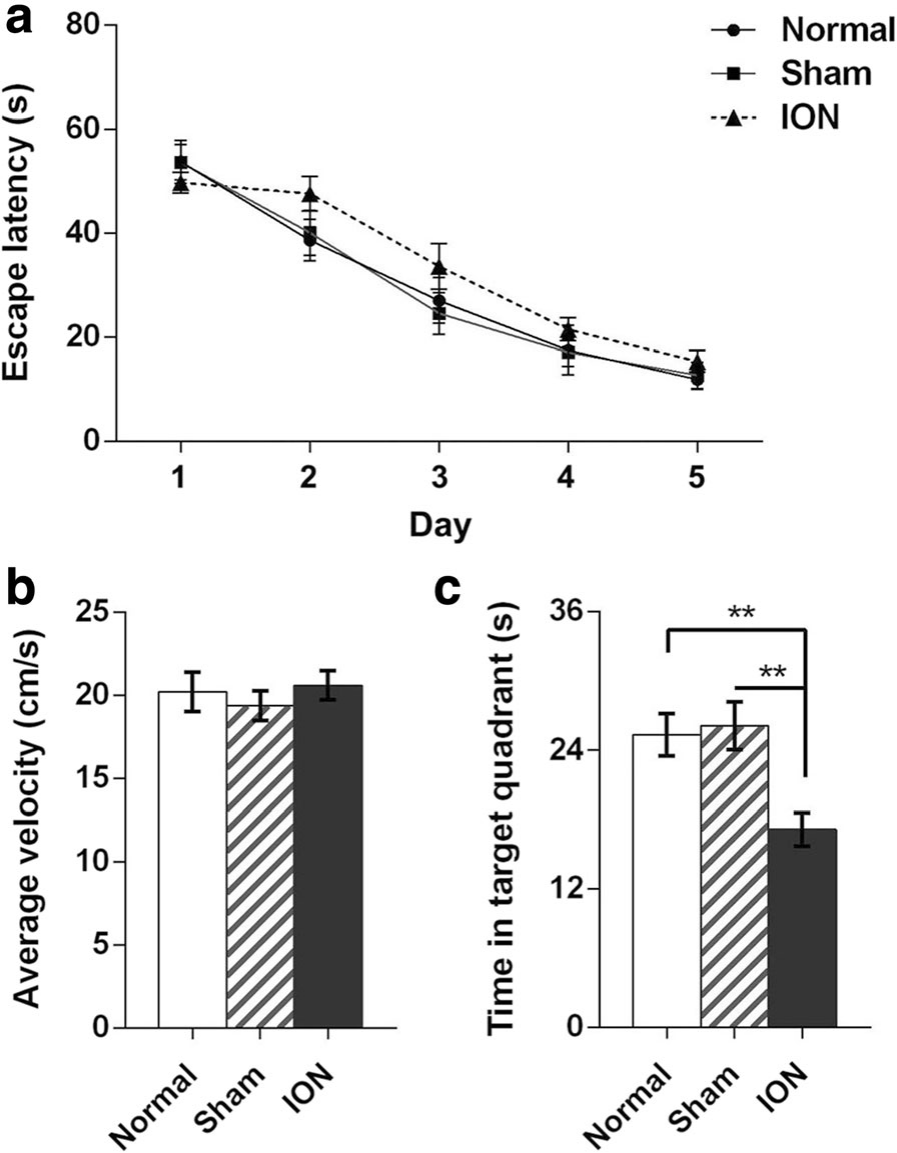
Behavioral data from Morris water maze test. **a** The escape latency to the platform over the 5 days during the acquisition phase was analyzed by repeated measures ANOVA. **b**, **c** Average velocity (**b**) and time in target quadrant during the probe trials (**c**) were analyzed by one-way ANOVA (**p < 0.01). Results are expressed as mean ± SEM. n = 8 (1:1 sex ratio) per group

During the acquisition phase of the Morris water maze test, mice were trained to recall the location of a hidden platform below the water surface. Figure 5 illustrates that all groups showed improved performance over the days (day: F_4,88_ = 68.591, *p* < 0.001) and no significant group effects were found in latency to reach the hidden platform in the training trials (group: F_2,22_ = 1.851, *p* = 0.181; group × day: F_8,88_ = 0.774, *p* = 0.627). Spatial memory of the platform location was examined in the probe phase during which the platform was removed from the pool and the mice were allowed to explore the pool for 1 min. The ION mice showed decreased time spent in the target quadrant where the platform was located (F_2,22_ = 8.10, *p* = 0.002; post hoc comparison, Normal to ION: p = 0.003; Sham to ION: *p* = 0.002; Fig. 5c). No significant differences were detected across the three groups in average velocity (F_2,22_ = 0.402, *p* = 0.674; Fig. 5b). The data sorted by sex are shown in Additional file 2: Table S1. The results showed that ION-transected mice have impaired spatial memory.

### Reduced hypothalamic OXT in ION-transected mice

**Fig. 6:**
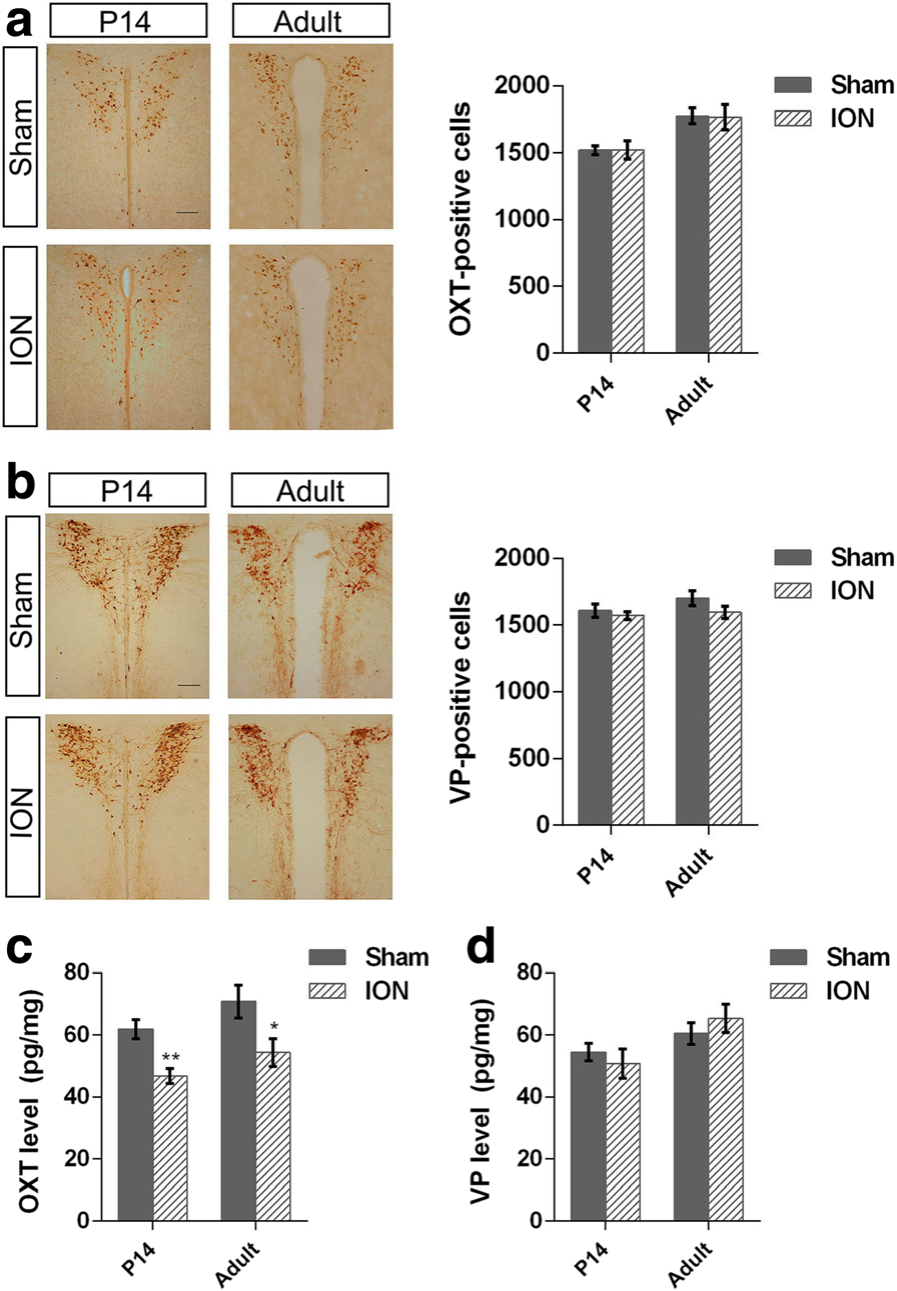
Immunoreactivity and peptide level of hypothalamic OXT and VP in P14 and adult mice. **a** Left: representative images of OXT immunoreactivity in the PVN. Scale bar, 100 μm. Right: number of OXT-positive neurons in the PVN. **b** Left: representative images of VP immunoreactivity in the PVN. Scale bar, 100 μm. Right: Number of VP-positive neurons in the PVN. **c** OXT peptide levels in the hypothalamus. **d** VP peptide levels in the hypothalamus. Results are expressed as mean ± SEM; *p < 0.05 via Student’s *t*-test. *n* = 4 (1:1 sex ratio) per group for (**a**, **b**); *n* = 6 (1:1 sex ratio) per group for (**c**, **d**)

Social memory is highly related to central OXT and vasopressin (VP) [29, 49], and a previous study has shown that reduction of OXT in pups with bilateral whisker deprivation from P0 to P14 [33]. We then examined their expressing levels in the hypothalamus by immunostaining and enzyme-linked immunoassay at P14 and in adult mice with unilateral ION transection at P3. There were no significant differences in the numbers of OXT- and VP-positive neurons in the PVN (OXT-positive cells: t_6_ = −0.01, *p* = 0.992 for P14, t_6_ = 0.082, *p* = 0.938 for adult, Fig. 6a; VP-positive cells: t_6_ = 0.623, *p* = 0.556 for P14, t_6_ = 1.432, *p* = 0.202 for adult, Fig. 6b), but OXT levels decreased significantly (t_10_ = 3.939, *p* = 0.003 for P14, t_10_ = 2.386, *p* = 0.038 for adult; Fig. 6c) in the ION mice compared with naive mice. VP levels, however, remained unchanged in ION mice (t_10_ = 0.685, *p* = 0.509 for P14, t_10_ = −0.888, *p* = 0.396 for adult; Fig. 6d). The results indicate that unilateral sensory deprivation by ION transection at P3 leads to a reduction of OXT levels in the hypothalamus, and this reduction is persisted into adulthood.

### Social memory of ION-transected mice is restored by OXT administration

**Fig. 7:**
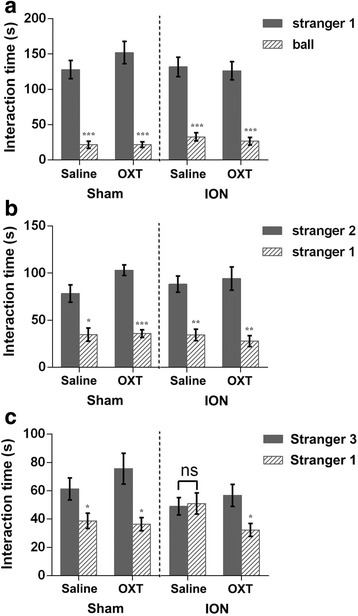
OXT administration improved social memory of ION mice. The three chamber test was conducted 20 min after OXT or saline administration. Intact sociability indicated by preference for a stranger mouse vs an inanimate ball (**a**) and social novelty revealed by preference for a novel stranger vs a familiar stranger (**b**) in ION-transected mice are not altered by nasal OXT administration. However, the social memory deficit in ION-transected mice shown by a similar investigation time to stranger 3 (*p* = 0.637) is largely restored by nasal OXT administration as shown by the appearance of spending more time with stranger 3 (**c**). Data were analyzed by repeated-measures ANOVA and results are expressed as mean ± SEM; *p < 0.05, **p < 0.01, ***p < 0.001 via Fisher’s PLSD post hoc tests. *n* = 8 (1:1 sex ratio) per group

The previous study has shown that OXT is an important mediator in social memory in rodents [50–52]. To examine if reduced OXT is involved in the social memory deficits, OXT was intranasally administered in ION-transected mice as reported previously [47, 48]. It has been shown that OXT level is increased in the brain with peak levels occurring 30–60 min after the nasal administration in mice [48], and it was confirmed by our data that OXT level was indeed increased at 30 min and 1 h after the administration (Additional file 3: Figure S2). We thus performed the three chamber test 20 min after the administration, which ensured our observation to be finished within this time window. The performances of control and ION-transected mice in interaction with stranger 1 and ball, and in interaction with stranger 1 and stranger 2 was not changed after the intranasal administration (All groups: treatment × side, *p* > 0.05; Fig. 7a, b). On the other hand, during the social memory phase, repeated-measures ANOVA revealed a significant interaction between treatment and side in ION-transected mice (ION: treatment × side: F_1,14_ = 5.906, *p* = 0.029; Fig. 7c). The ION mice treated with OXT spent more time investigating the novel stranger 3 and less time investigating the familiar stranger 1 compared with those treated with saline, suggesting that social memory is improved significantly. Fisher’s PLSD post hoc analysis revealed that only the ION group treated with saline showed reduced social memory, as observed in the similar investigation time spent with novel stranger 3 compared with the familiar stranger 1 (Sham with saline: F_1,7_ = 9.897, *p* < 0.05; Sham with OXT: F_1,7_ = 11.431, *p* < 0.05; ION with saline: F_1,7_ = 0.241, *p* > 0.05; ION with OXT: F_1,7_ = 5.860, *p* < 0.05). These results illustrate that the social memory deficit of ION-transected mice can be significantly restored by intranasal OXT administration.

## Discussion

It is well known that sensory perception and exploration, especially during the early perinatal period, strongly promotes cortical development and functional maturation. This process is, however, extremely vulnerable to deleterious effects including sensory deprivation. In mice, transection of ION can lead to loss of barrel structures and callosal connection in the somatosensory cortex [5, 6, 22]. However, few studies have explored behavioral alterations beyond the whisker-related somatosensory system in these mice. In the present study, we utilized mice with unilateral transections of ION at P3 and determined their behavioral alterations in adulthood. ION-transected mice showed intact locomotor activity, motor coordination, olfaction, basal anxiety-like behaviors, novel object memory, preference for social novelty, and sociability. However, spatial memory and social memory were compromised in ION-transected mice.

Barrels in layer IV of the somatosensory cortex are the cortical correlates of contralateral whiskers, and neurons within each barrel respond best to a particular whisker on the contralateral face [53, 54]. During early postnatal development, the formation of barrels represents a condensation of individual whisker-related thalamocortical axons and rearrangement of cortical neurons in corresponding barrel regions [55]. The alterations in the brain caused by the early sensory deprivation are not limited to loss of barrels. For example, the early sensory deprivation also affects synaptic plasticity [14, 15], dendritic spine refinement and formation of local circuitry [16–20], and inter-hemispheric in the somatosensory cortex [22]. We thus speculated the spatial memory and social memory deficits in ION-transected mice are the consequence of global structural and functional alterations in brain circuits after the early sensory deprivation.

Morris water maze is widely used in accessing spatial learning and memory of rodents since it was introduced [56], and the hippocampus is a key brain region for it [57, 58]. Our previous studies have shown that early unilateral sensory deprivation impairs the formation of callosal projection between the bilateral somatosensory cortices [22]. Corpus callosum is involved in the communication of two hemispheres, which has been evidenced in patients with callosotomy (split-brain) by examination of latererized brain functions in dominant hemisphere [59, 60]; these higher brain functions could not examined in rodents. However, an interesting study has reported impaired contextual fear memory in split-brain mice [61], an inbred mouse strain displaying agenesis of the corpus callosum and reduced hippocampal commissure [61] and mimicking patients with split brains. Like spatial memory, contextual fear memory is also hippocampus dependent. Considering similarities in the callosal connections and behavioral phenotypes between the split-brain mice and ION-transected mice, we suggest that impaired spatial memory in ION-transected mice may be caused by defective callosal connections [22] along with unknown structural and functional alterations after early sensory deprivation.

It has been proposed that a balanced activity of OXT and VP systems is important for appropriate emotional behaviors, and shifting the balance towards oxytocin may help to improve emotional behaviors [62], and OXT is an important mediator in social memory as shown by an increase of OXT release from during the retrieval of social memory and impaired social memory by blocking OXT activity with OXT receptor antagonist [52]. To explore the mechanism underlying defective social memory phenotype, we assessed the levels of these two neuropeptides in the hypothalamus, where the two peptides are synthesized. ION-transected mice showed a reduction of OXT without significant changes of VP. It is likely that balanced activity of OXT and VP systems may be disrupted in ION-transected mice thereby contributing to the social memory deficits. In support of this, the social memory deficit of ION-transected mice was significantly restored by intranasal administration of OXT. It should be noted it is unclear the reduced OXT level in the brain is caused by a decrease of synthesis or an increase of release or both of them. The previous study has shown that lowered OXT levels in the brain is caused by reduced synthesis shown by lowered OXT mRNA level in pups with bilateral sensory deprivation [33].

It has been shown that deficient OXT system is implicated in the development of autism and supplement of OXT in the brain by intranasal administration of OXT increases retention of social cognition in patients with autism [63–65]. The new DSM-5 diagnostic criteria published in 2013 include sensory problems as part of the core symptoms of autism spectrum disorders. Most children with autism exhibit sensory processing dysfunctions and hypersensitivity or hyposensitivity in response to selective sensory stimuli [66]. Sensory experience in early life is necessary for the development of proper multisensory processing [67, 68] and this cross-model plasticity is mediated by OXT [33]. Thus, a link between deficient OXT system and autism have been established [29, 69], and in support of this, OXT knockout mice show reduced social memory [28], an important behavioral phenotype of autism [70]. Taken together, we can infer that altered sensory inputs in early life may be a potential etiological factor of autism, and if so, OXT may be considered a crucial regulator in this scenario.

In the present study, we provide evidence that that adult mice with unilateral transection of ION at P3 display deficits in social memory and spatial memory. We also demonstrate that the social memory deficit in ION-transected mice is likely caused by lowered OXT level in the brain.

## Additional files

Additional file 1: Figure S1. Loss of barrels in the contralateral somatosensory cortex after unilateral transection of ION at P3. Nissl staining shows that, barrels (arrows) are clearly seen in bilateral cortices of sham-operated adult mice, whereas they are not detectable in the contralateral cortex in P3 ION-transected mice. Scale bars, 250 μm. (JPG 85 kb)
Additional file 2: Table S1. Gender effects in the behavioral tests. (DOCX 48 kb)
Additional file 3: Figure S2. OXT levels in the brain is elevated after intranasal administration of OXT. (JPG 919 kb)

## Abbreviations

DAB: Diaminobenzidine
DLE: Dark-light exploration test
EIA: Enzyme-linked immunoassay analyses
EPM: Elevated plus maze test
ION: Infraorbital nerve
MWM: Morris water maze test
NOR: Novel object recognition test
OF: Open field test
OXT: Oxytocin
P: Postnatal day
PBS: Phosphate-buffered saline
PFA: Paraformaldehyde
PVN: Hypothalamic paraventricular nuclei
VP: Vasopressin
