# Supplementary figures and images for: Oxytocin is implicated in social memory deficits induced by early sensory deprivation in mice
Source: Mol Brain. 2016 Dec 13;9:98. doi: 10.1186/s13041-016-0278-3 (PMC5155398; doi:10.1186/s13041-016-0278-3)

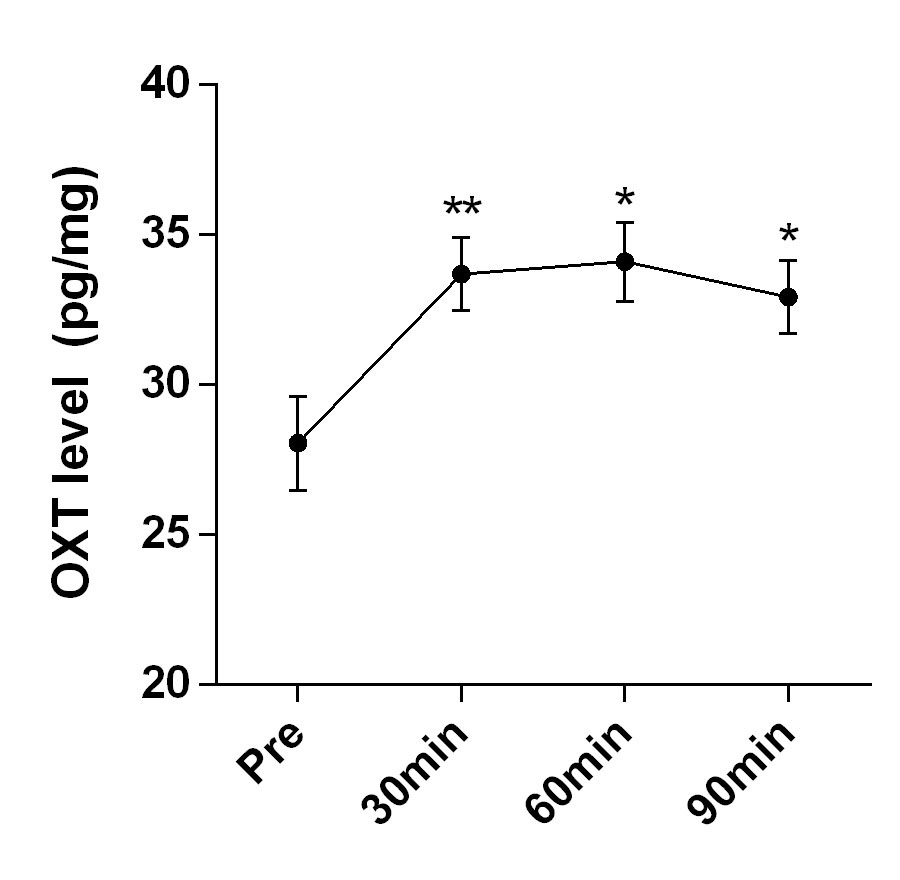

Supplement: Additional file 1: Figure S1. — Loss of barrels in the contralateral somatosensory cortex after unilateral transection of ION at P3. Nissl staining shows that, barrels (arrows) are clearly seen in bilateral cortices of sham-operated adult mice, whereas they are not detectable in the contralateral cortex in P3 ION-transected mice. Scale bars, 250 μm. (JPG 85 kb) [file 13041_2016_278_MOESM1_ESM.jpg]

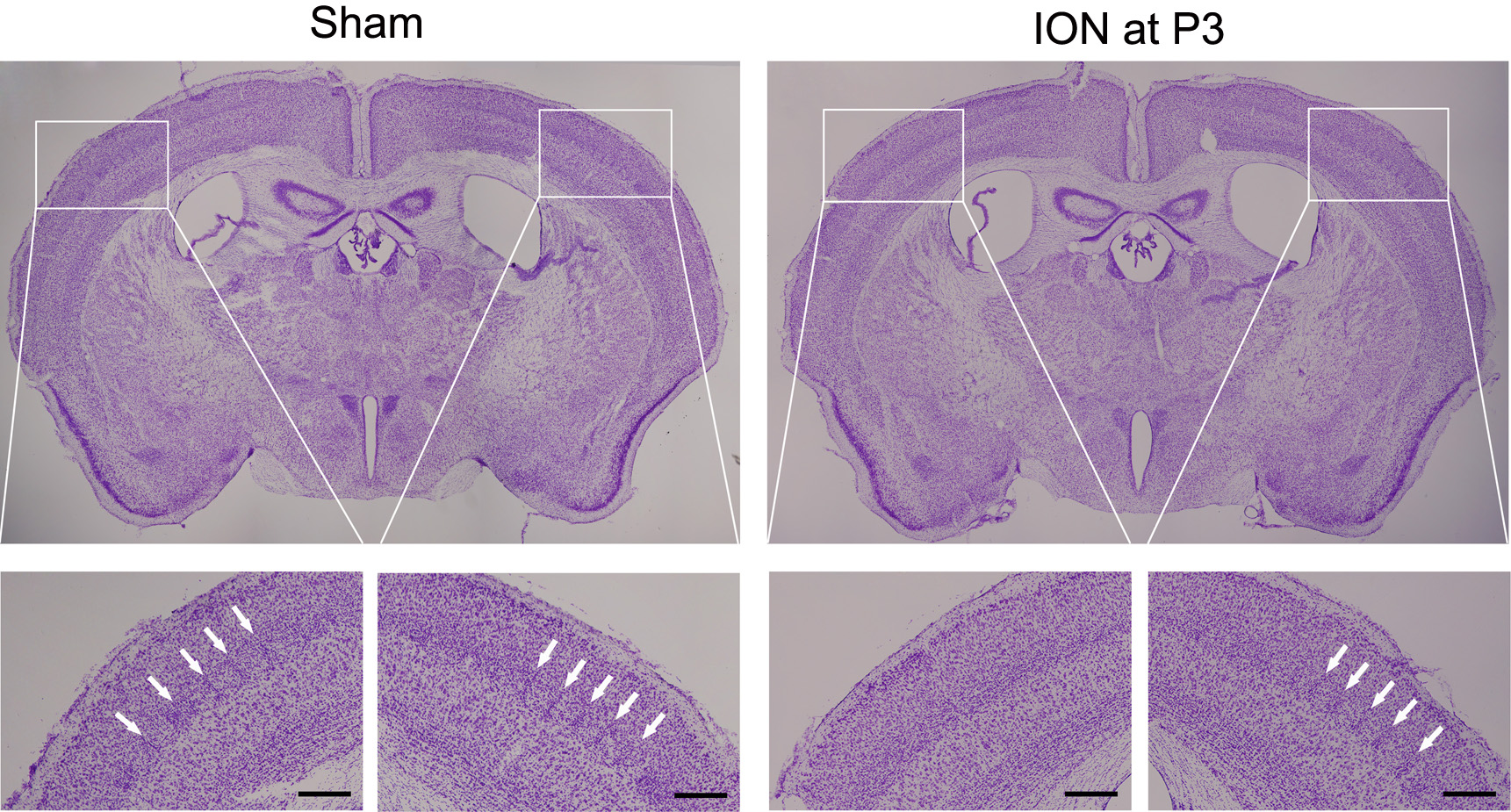

Supplement: Additional file 3: Figure S2. — OXT levels in the brain is elevated after intranasal administration of OXT. (JPG 919 kb) [file 13041_2016_278_MOESM3_ESM.jpg]
