# Supplementary material for: Oxytocin is implicated in social memory deficits induced by early sensory deprivation in mice
Source: Mol Brain. 2016 Dec 13;9:98. doi: 10.1186/s13041-016-0278-3 (PMC5155398; doi:10.1186/s13041-016-0278-3)
Supplement: Additional file 2: Table S1. — Gender effects in the behavioral tests. (DOCX 48 kb) [file 13041_2016_278_MOESM2_ESM.docx]

Additional file 2: Table S1.

Gender effects in the behavioral tests

| *Open field test* | | | | | | | | | | | |
| --- | --- | --- | --- | --- | --- | --- | --- | --- | --- | --- | --- |
| Measurement | Gender | Group | Mean | | SEM | | N | Statistical test | p value | Statistical test | p value |
| Average velocity (cm/s) | Male | Sham | 39.77 | | 0.97 | | 5 | Two-way ANOVA | n.s. | Unpaired two-tailed t-test | n.s. |
|  |  | ION | 37.44 | | 1.42 | | 6 |  |  |  |  |
|  | Female | Sham | 41.66 | | 1.57 | | 8 |  |  | Unpaired two-tailed t-test | n.s. |
|  |  | ION | 41.70 | | 1.90 | | 7 |  |  |  |  |
| Distance traveled (cm) | Male | Sham | 5773.44 | | 605.60 | | 5 | Two-way ANOVA | n.s. | Unpaired two-tailed t-test | n.s. |
|  |  | ION | 4991.25 | | 382.13 | | 6 |  |  |  |  |
|  | Female | Sham | 5773.50 | | 387.52 | | 8 |  |  | Unpaired two-tailed t-test | n.s. |
|  |  | ION | 5456.62 | | 364.42 | | 7 |  |  |  |  |
| Distance traveled in center (cm) | Male | Sham | 719.14 | | 79.79 | | 5 | Two-way ANOVA | n.s. | Unpaired two-tailed t-test | n.s. |
|  |  | ION | 597.50 | | 89.92 | | 6 |  |  |  |  |
|  | Female | Sham | 711.81 | | 78.26 | | 8 |  |  | Unpaired two-tailed t-test | n.s. |
|  |  | ION | 728.57 | | 64.61 | | 7 |  |  |  |  |
| Time in center (s) | Male | Sham | 107.49 | | 19.98 | | 5 | Two-way ANOVA | n.s. | Unpaired two-tailed t-test | n.s. |
|  |  | ION | 84.88 | | 14.41 | | 6 |  |  |  |  |
|  | Female | Sham | 111.9 | | 12.33 | | 8 |  |  | Unpaired two-tailed t-test | n.s. |
|  |  | ION | 135.1 | | 30.32 | | 7 |  |  |  |  |
| *Novel object recognition test* | | | | | | | | | | | |
| Discrimination index | Male | Sham | 0.36 | | 0.04 | | 5 | Two-way ANOVA | n.s. | Unpaired two-tailed t-test | n.s. |
|  |  | ION | 0.34 | | 0.03 | | 6 |  |  |  |  |
|  | Female | Sham | 0.36 | | 0.04 | | 6 |  |  | Unpaired two-tailed t-test | n.s. |
|  |  | ION | 0.40 | | 0.04 | | 6 |  |  |  |  |
| *Dark/light exploration test* | | | | | | | | | | | |
| Entries into light box | Male | Sham | 8.00 | | 1.125 | | 6 | Two-way ANOVA | n.s. | Unpaired two-tailed t-test | n.s. |
|  |  | ION | 9.20 | | 1.66 | | 5 |  |  |  |  |
|  | Female | Sham | 8.83 | | 1.49 | | 6 |  |  | Unpaired two-tailed t-test | n.s. |
|  |  | ION | 8.14 | | 1.60 | | 7 |  |  |  |  |
| Time in light box (s) | Male | Sham | 54.75 | | 20.10 | | 6 | Two-way ANOVA | n.s. | Unpaired two-tailed t-test | n.s. |
|  |  | ION | 46.92 | | 8.18 | | 5 |  |  |  |  |
|  | Female | Sham | 58.74 | | 16.17 | | 6 |  |  | Unpaired two-tailed t-test | n.s. |
|  |  | ION | 52.14 | | 12.38 | | 7 |  |  |  |  |
| *Elevated plus maze test* | | | | | | | | | | | |
| Open arm entries | Male | Sham | 12.50 | | 1.46 | | 6 | Two-way ANOVA | n.s. | Unpaired two-tailed t-test | n.s. |
|  |  | ION | 10.60 | | 1.12 | | 5 |  |  |  |  |
|  | Female | Sham | 12.17 | | 1.58 | | 6 |  |  | Unpaired two-tailed t-test | n.s. |
|  |  | ION | 13.86 | | 1.08 | | 7 |  |  |  |  |
| Time in open arms (s) | Male | Sham | 54.42 | | 10.85 | | 6 | Two-way ANOVA | n.s. | Unpaired two-tailed t-test | n.s. |
|  |  | ION | 48.64 | | 8.08 | | 5 |  |  |  |  |
|  | Female | Sham | 37.60 | | 7.33 | | 6 |  |  | Unpaired two-tailed t-test | n.s. |
|  |  | ION | 57.35 | | 7.62 | | 7 |  |  |  |  |
| *Two-trial direct interaction test* | | | | | | | | | | | |
| Measurement | Gender | Group | Trials | Mean | | SEM | N | Statistical test | P value | Statistical test | P value |
| Interaction time with same stimuli (s) | Male | Sham | Trial 1 | 60.94 | | 7.38 | 5 | Repeated measures ANOVA | Trial  <0.001  Group  <0.01  Group*Trial  <0.01 | Paired samples t-test | <0.05 |
|  |  |  | Trial 2 | 25.61 | | 7.80 | 5 |  |  |  |  |
|  |  | ION | Trial 1 | 56.95 | | 6.70 | 6 |  |  | Paired samples t-test | n.s. |
|  |  |  | Trial 2 | 44.55 | | 4.39 | 6 |  |  |  |  |
|  | Female | Sham | Trial 1 | 45.29 | | 3.87 | 7 |  |  | Paired samples t-test | <0.01 |
|  |  |  | Trial 2 | 24.01 | | 2.90 | 7 |  |  |  |  |
|  |  | ION | Trial 1 | 49.49 | | 3.94 | 6 |  |  | Paired samples t-test | n.s. |
|  |  |  | Trial 2 | 47.96 | | 4.28 | 6 |  |  |  |  |
| Interaction time with novel stimuli (s) | Male | Sham | Trial 1 | 69.31 | | 5.08 | 4 | Repeated measures ANOVA | Gender  <0.05  Gender*Group  <0.05 | Paired samples t-test | n.s. |
|  |  |  | Trial 2 | 60.87 | | 6.84 | 4 |  |  |  |  |
|  |  | ION | Trial 1 | 48.49 | | 5.21 | 4 |  |  | Paired samples t-test | n.s. |
|  |  |  | Trial 2 | 50.57 | | 4.53 | 4 |  |  |  |  |
|  | Female | Sham | Trial 1 | 39.95 | | 3.04 | 4 |  |  | Paired samples t-test | n.s. |
|  |  |  | Trial 2 | 36.62 | | 9.26 | 4 |  |  |  |  |
|  |  | ION | Trial 1 | 58.19 | | 5.18 | 4 |  |  | Paired samples t-test | n.s. |
|  |  |  | Trial 2 | 42.51 | | 1.71 | 4 |  |  |  |  |
| *Three chamber test* | | | | | | | | | | | |
| Measurement | Gender | Group | Side | Mean | | SEM | N | Statistical test | P value | Statistical test | P value |
| Interaction time in sociability test (s) | Male | Sham | Stranger 1 | 78.52 | | 15.28 | 5 | Repeated measures ANOVA | Side  <0.001 | Paired samples t-test | <0.05 |
|  |  |  | Ball | 20.40 | | 7.34 | 5 |  |  |  |  |
|  |  | ION | Stranger 1 | 74.73 | | 7.07 | 6 |  |  | Paired samples t-test | <0.01 |
|  |  |  | Ball | 11.68 | | 3.74 | 6 |  |  |  |  |
|  | Female | Sham | Stranger 1 | 87.93 | | 19.11 | 6 |  |  | Paired samples t-test | <0.05 |
|  |  |  | Ball | 22.38 | | 4.21 | 6 |  |  |  |  |
|  |  | ION | Stranger 1 | 108.81 | | 13.34 | 6 |  |  | Paired samples t-test | <0.01 |
|  |  |  | Ball | 18.97 | | 5.99 | 6 |  |  |  |  |
| Interaction time in social novelty test (s) | Male | Sham | Stranger 2 | 52.54 | | 4.68 | 5 | Repeated measures ANOVA | Side  <0.001 | Paired samples t-test | <0.05 |
|  |  |  | Stranger 1 | 20.69 | | 8.87 | 5 |  |  |  |  |
|  |  | ION | Stranger 2 | 69.37 | | 13.18 | 6 |  |  | Paired samples t-test | <0.05 |
|  |  |  | Stranger 1 | 12.88 | | 3.24 | 6 |  |  |  |  |
|  | Female | Sham | Stranger 2 | 64.28 | | 4.27 | 6 |  |  | Paired samples t-test | <0.01 |
|  |  |  | Stranger 1 | 20.36 | | 5.72 | 6 |  |  |  |  |
|  |  | ION | Stranger 2 | 77.51 | | 15.43 | 6 |  |  | Paired samples t-test | <0.05 |
|  |  |  | Stranger 1 | 22.42 | | 6.24 | 6 |  |  |  |  |
| Interaction time in social memory test (s) | Male | Sham | Stranger 3 | 57.62 | | 9.20 | 5 | Repeated measures ANOVA | Side  <0.001  Group*Side  <0.001  Gender*Group*Side  <0.05 | Paired samples t-test | <0.05 |
|  |  |  | Stranger 1 | 31.08 | | 4.90 | 5 |  |  |  |  |
|  |  | ION | Stranger 3 | 41.68 | | 4.51 | 6 |  |  | Paired samples t-test | <0.05 |
|  |  |  | Stranger 1 | 29.16 | | 3.30 | 6 |  |  |  |  |
|  | Female | Sham | Stranger 3 | 57.14 | | 6.95 | 6 |  |  | Paired samples t-test | <0.01 |
|  |  |  | Stranger 1 | 19.67 | | 4.73 | 6 |  |  |  |  |
|  |  | ION | Stranger 3 | 38.00 | | 4.17 | 6 |  |  | Paired samples t-test | n.s. |
|  |  |  | Stranger 1 | 43.99 | | 5.40 | 6 |  |  |  |  |
| *ROTA-ROD* | | | | | | | | | | | |
| Measurement | Gender | Group | Day | Mean | | SEM | N | Statistical test | P value | Statistical test | P value |
| Latency to fall (s) | Male | Sham | Day 1 | 184.67 | | 23.82 | 6 | Repeated measures ANOVA | Day  <0.001 | - | - |
|  |  |  | Day 2 | 221.58 | | 24.64 | 6 |  |  |  |  |
|  |  |  | Day 3 | 224.67 | | 22.28 | 6 |  |  |  |  |
|  |  |  | Day 4 | 252.00 | | 16.85 | 6 |  |  |  |  |
|  |  |  | Day 5 | 266.17 | | 10.85 | 6 |  |  |  |  |
|  |  | ION | Day 1 | 172.36 | | 17.70 | 7 |  |  |  |  |
|  |  |  | Day 2 | 220.50 | | 20.95 | 7 |  |  |  |  |
|  |  |  | Day 3 | 212.79 | | 12.95 | 7 |  |  |  |  |
|  |  |  | Day 4 | 220.43 | | 11.53 | 7 |  |  |  |  |
|  |  |  | Day 5 | 262.21 | | 15.05 | 7 |  |  |  |  |
|  | Female | Sham | Day 1 | 185.58 | | 18.13 | 6 |  |  | - | - |
|  |  |  | Day 2 | 190.00 | | 11.22 | 6 |  |  |  |  |
|  |  |  | Day 3 | 251.50 | | 12.05 | 6 |  |  |  |  |
|  |  |  | Day 4 | 251.67 | | 14.46 | 6 |  |  |  |  |
|  |  |  | Day 5 | 274.75 | | 11.46 | 6 |  |  |  |  |
|  |  | ION | Day 1 | 181.60 | | 34.23 | 5 |  |  |  |  |
|  |  |  | Day 2 | 213.70 | | 18.52 | 5 |  |  |  |  |
|  |  |  | Day 3 | 220.80 | | 23.59 | 5 |  |  |  |  |
|  |  |  | Day 4 | 255.40 | | 14.21 | 5 |  |  |  |  |
|  |  |  | Day 5 | 268.50 | | 12.02 | 5 |  |  |  |  |
| *Olfactory habituation/dishabituation test* | | | | | | | | | | | |
| Measurement | Gender | Group | Odor | Mean | | SEM | N | Statistical test | P value | Statistical test | P value |
| Time spent sniffing (s) | Male | Sham | Water 1 | 13.70 | | 4.24 | 5 | Repeated measures ANOVA  ( for water,  orange,  and urine respectively) | Water  <0.001  Orange  <0.001  Urine  <0.001 | - | - |
|  |  |  | Water 2 | 5.13 | | 1.22 | 5 |  |  |  |  |
|  |  |  | Water 3 | 5.23 | | 1.47 | 5 |  |  | Paired samples t-test  (W3 vs. O1) | n.s. |
|  |  |  | Orange 1 | 15.33 | | 5.63 | 5 |  |  |  |  |
|  |  |  | Orange 2 | 3.20 | | 1.41 | 5 |  |  | - | - |
|  |  |  | Orange 3 | 2.14 | | 1.09 | 5 |  |  | Paired samples t-test  (O3 vs. U1) | <0.05 |
|  |  |  | Urine 1 | 15.86 | | 2.93 | 5 |  |  |  |  |
|  |  |  | Urine 2 | 7.37 | | 1.94 | 5 |  |  | - | - |
|  |  |  | Urine 3 | 3.08 | | 1.28 | 5 |  |  |  |  |
|  |  | ION | Water 1 | 11.01 | | 1.95 | 6 |  |  |  |  |
|  |  |  | Water 2 | 5.01 | | 0.80 | 6 |  |  |  |  |
|  |  |  | Water 3 | 2.93 | | 0.60 | 6 |  |  | Paired samples t-test  (W3 vs. O1) | n.s. |
|  |  |  | Orange 1 | 14.40 | | 4.74 | 6 |  |  |  |  |
|  |  |  | Orange 2 | 4.30 | | 0.44 | 6 |  |  | - | - |
|  |  |  | Orange 3 | 1.95 | | 0.43 | 6 |  |  | Paired samples t-test  (O3 vs. U1) | <0.01 |
|  |  |  | Urine 1 | 23.02 | | 4.78 | 6 |  |  |  |  |
|  |  |  | Urine 2 | 4.63 | | 1.51 | 6 |  |  | - | - |
|  |  |  | Urine 3 | 3.59 | | 0.80 | 6 |  |  |  |  |
|  | Female | Sham | Water 1 | 10.26 | | 1.57 | 6 |  |  |  |  |
|  |  |  | Water 2 | 4.70 | | 1.43 | 6 |  |  |  |  |
|  |  |  | Water 3 | 3.31 | | 1.09 | 6 |  |  | Paired samples t-test  (W3 vs. O1) | <0.05 |
|  |  |  | Orange 1 | 16.44 | | 3.55 | 6 |  |  |  |  |
|  |  |  | Orange 2 | 5.34 | | 1.06 | 6 |  |  | - | - |
|  |  |  | Orange 3 | 2.23 | | 0.76 | 6 |  |  | Paired samples t-test  (O3 vs. U1) | <0.01 |
|  |  |  | Urine 1 | 20.77 | | 2.87 | 6 |  |  |  |  |
|  |  |  | Urine 2 | 4.81 | | 0.77 | 6 |  |  | - | - |
|  |  |  | Urine 3 | 1.91 | | 0.37 | 6 |  |  |  |  |
|  |  | ION | Water 1 | 11.05 | | 1.03 | 6 |  |  |  |  |
|  |  |  | Water 2 | 6.09 | | 1.19 | 6 |  |  |  |  |
|  |  |  | Water 3 | 2.66 | | 0.78 | 6 |  |  | Paired samples t-test  (W3 vs. O1) | <0.01 |
|  |  |  | Orange 1 | 13.98 | | 1.29 | 6 |  |  |  |  |
|  |  |  | Orange 2 | 5.37 | | 1.33 | 6 |  |  | - | - |
|  |  |  | Orange 3 | 1.64 | | 0.26 | 6 |  |  | Paired samples t-test  (O3 vs. U1) | <0.001 |
|  |  |  | Urine 1 | 20.38 | | 1.53 | 6 |  |  |  |  |
|  |  |  | Urine 2 | 5.85 | | 1.09 | 6 |  |  | - | - |
|  |  |  | Urine 3 | 1.61 | | 0.46 | 6 |  |  |  |  |
| *Morris water maze test* | | | | | | | | | | | |
| Measurement | Gender | Group | Day | Mean | | SEM | N | Statistical test | P value | Statistical test | P value |
| Escape latency (s) | Male | Sham | Day 1 | 52.07 | | 7.02 | 4 | Repeated measures ANOVA | Day  <0.001  Gender*Day  <0.05 | - | - |
|  |  |  | Day 2 | 40.73 | | 5.06 | 4 |  |  |  |  |
|  |  |  | Day 3 | 18.62 | | 2.54 | 4 |  |  |  |  |
|  |  |  | Day 4 | 19.00 | | 4.58 | 4 |  |  |  |  |
|  |  |  | Day 5 | 16.52 | | 3.67 | 4 |  |  |  |  |
|  |  | ION | Day 1 | 50.83 | | 3.67 | 5 |  |  |  |  |
|  |  |  | Day 2 | 47.99 | | 5.44 | 5 |  |  |  |  |
|  |  |  | Day 3 | 28.28 | | 3.76 | 5 |  |  |  |  |
|  |  |  | Day 4 | 20.46 | | 2.56 | 5 |  |  |  |  |
|  |  |  | Day 5 | 19.71 | | 1.60 | 5 |  |  |  |  |
|  | Female | Sham | Day 1 | 55.23 | | 1.73 | 4 |  |  | - | - |
|  |  |  | Day 2 | 39.43 | | 7.89 | 4 |  |  |  |  |
|  |  |  | Day 3 | 30.52 | | 6.65 | 4 |  |  |  |  |
|  |  |  | Day 4 | 15.14 | | 3.37 | 4 |  |  |  |  |
|  |  |  | Day 5 | 8.85 | | 2.56 | 4 |  |  |  |  |
|  |  | ION | Day 1 | 48.40 | | 0.59 | 4 |  |  |  |  |
|  |  |  | Day 2 | 47.14 | | 4.30 | 4 |  |  |  |  |
|  |  |  | Day 3 | 40.30 | | 8.24 | 4 |  |  |  |  |
|  |  |  | Day 4 | 22.86 | | 4.29 | 4 |  |  |  |  |
|  |  |  | Day 5 | 10.00 | | 1.88 | 4 |  |  |  |  |
| Measurement | Gender | Group | Mean | | SEM | | N | Statistical test | P value | Statistical test | P value |
| Average velocity (cm/s) | Male | Sham | 18.35 | | 1.68 | | 4 | Two-way ANOVA | n.s. | Unpaired two-tailed t-test | n.s. |
|  |  | ION | 20.72 | | 1.61 | | 5 |  |  |  |  |
|  | Female | Sham | 20.39 | | 0.52 | | 4 |  |  | Unpaired two-tailed t-test | n.s. |
|  |  | ION | 20.46 | | 0.64 | | 4 |  |  |  |  |
| Time in target quadrant (s) | Male | Sham | 27.70 | | 3.19 | | 4 | Two-way ANOVA | Group  <0.01 | Unpaired two-tailed t-test | <0.05 |
|  |  | ION | 16.20 | | 1.95 | | 5 |  |  |  |  |
|  | Female | Sham | 24.55 | | 1.73 | | 4 |  |  | Unpaired two-tailed t-test | n.s. |
|  |  | ION | 18.35 | | 2.30 | | 4 |  |  |  |  |
